# Bacterial Pathogen Emergence Requires More than Direct Contact with a Novel Passerine Host

**DOI:** 10.1128/IAI.00863-17

**Published:** 2018-02-20

**Authors:** Molly Staley, Geoffrey E. Hill, Chloe C. Josefson, Jonathan W. Armbruster, Camille Bonneaud

**Affiliations:** a Department of Biological Sciences, Auburn University, Auburn, Alabama, USA; b Auburn University Museum of Natural History, Auburn University, Auburn, Alabama, USA; c Biosciences, University of Exeter, Penryn Cornwall, United Kingdom; Washington State University

**Keywords:** Mycoplasma gallisepticum, disease emergence, host shift

## Abstract

While direct contact may sometimes be sufficient to allow a pathogen to jump into a new host species, in other cases, fortuitously adaptive mutations that arise in the original donor host are also necessary. Viruses have been the focus of most host shift studies, so less is known about the importance of ecological versus evolutionary processes to successful bacterial host shifts. Here we tested whether direct contact with the novel host was sufficient to enable the mid-1990s jump of the bacterium Mycoplasma gallisepticum from domestic poultry to house finches (Haemorhous mexicanus). We experimentally inoculated house finches with two genetically distinct M. gallisepticum strains obtained either from poultry (Rlow) or from house finches (HF1995) during an epizootic outbreak. All 15 house finches inoculated with HF1995 became infected, whereas Rlow successfully infected 12 of 15 (80%) inoculated house finches. Comparisons among infected birds showed that, relative to HF1995, Rlow achieved substantially lower bacterial loads in the host respiratory mucosa and was cleared faster. Furthermore, Rlow-infected finches were less likely to develop clinical symptoms than HF1995-infected birds and, when they did, displayed milder conjunctivitis. The lower infection success of Rlow relative to HF1995 was not, however, due to a heightened host antibody response to Rlow. Taken together, our results indicate that contact between infected poultry and house finches was not, by itself, sufficient to explain the jump of M. gallisepticum to house finches. Instead, mutations arising in the original poultry host would have been necessary for successful pathogen emergence in the novel finch host.

## INTRODUCTION

Recent outbreaks of novel diseases in humans and domestic animals underscore the critical need to elucidate the factors that enable pathogens to become established in new host species (1–5). Host shifts require not only that pathogens come in direct contact with the novel host but also that they have the capacity to infect and be transmitted by the new host (6, 7). Contact depends on opportunities for the pathogen to leave the original host and gain access to a novel host and, because of this, is mitigated by the geographic ranges and ecologies of both the hosts and the pathogen (8). For instance, exposure of European rabbits (Oryctolagus cuniculus) to the myxoma virus during an eradication attempt in Australia in the mid-20th century was sufficient to allow for pathogen emergence, even though the virus' natural host is a South American leporid rabbit (Sylvagus brasiliensis) (9, 10). Infectiousness and transmission, in contrast, will be determined primarily by pathogen and host genotypes (11, 12). For example, humans have long been in contact with pathogens of Himalayan palm civets (Paguma larvata), which are traditional food items in China. Despite this, there had been no known host shifts from civets to humans until the emergence of the severe acute respiratory syndrome (SARS) virus in 2002, then made possible by adaptive genetic changes in the virus' receptor binding domain (13, 14). The extent to which hosts shifts are limited by opportunities for contact with novel hosts versus by fortuitous mutations that predispose the pathogen to infect the novel host remains, however, understudied despite the potential impacts on humans and livestock of host shifts by pathogens.

In recent decades, molecular analyses have revealed that host shifting by bacterial pathogens may occur more frequently than previously thought (15–18). For example, phylogenetic analyses suggest that Wolbachia bacteria independently colonized multiple species of arthropods via horizontal transmission (15, 19, 20). Staphylococcus aureus similarly exhibits a diverse host range, including poultry, ruminants, and other mammals, likely the result of host shifting from humans (17, 18). Indeed, the jump of S. aureus from humans to rabbits required only a single mutation in a gene encoding an integral membrane protein (1). Yet, for other bacteria, there is evidence of more restrictive host ranges despite regular contact with other potential host species. For example, wood mice (Apodemus sylvaticus) and bank voles (Myoedes glareolus) in the United Kingdom harbor unique variants of Bartonella despite the collection of fleas carrying bank vole-specific variants from wood mice and vice versa (21). Experimental studies on cotton rats (Sigmodon hispidus) and white-footed mice (Peromyscus leucopus) similarly found that Bartonella infections were successful only when bacteria originated from the same host species or from their close phylogenetic relatives (22). An important limitation to understanding the role of contact versus host suitability in bacterial host shifts is that a majority of studies have focused on viral pathogens (11). Yet bacterial host shifts may be subject to different constraints than viral host shifts; unlike viruses, bacteria must also be able to extract essential metabolic substrates, nutrients, and enzymatic cofactors, such as iron, from their host and may face a suite of different host immune defenses (23). As a result, further studies are required to better understand the role of ecological versus evolutionary factors in bacterial host shifts.

One notable host shift by a bacterial pathogen occurred when Mycoplasma gallisepticum emerged in eastern North American house finches (Haemorhous mexicanus) in 1994. Comparative genomic analyses confirmed that this epizootic, which caused measurable declines in eastern U.S. house finch populations, resulted from a single host shift event of M. gallisepticum from poultry that occurred in the mid-1990s (2, 24–28). The subsequent spread of M. gallisepticum throughout North American house finches was uniquely well documented thanks to externally visible symptoms of conjunctivitis, quick identification of M. gallisepticum as the causative agent, and active disease monitoring (2, 24). Since then, spillover infections have been documented in numerous other wild bird species (29, 30), although none have led to an epizootic-scale outbreak, the reason for which remains unclear.

To investigate whether direct contact was sufficient for M. gallisepticum to jump into house finches, we experimentally inoculated house finches either with an M. gallisepticum strain obtained from the original poultry host (Rlow) or with a strain collected during the epizootic outbreak in the novel house finch host (HF1995) (26, 30–32). Whole-genome comparisons have revealed that HF1995 exhibits widespread genomic changes compared to Rlow (26), but the functional significance of these genomic changes for colonizing the novel host remains unknown. We predicted that if contact with the novel host alone was sufficient for M. gallisepticum to infect house finches, then Rlow and HF1995 should display similar abilities to establish an infection and cause clinical disease in house finches. Conversely, if Rlow showed low or no capacity to infect house finches, then this would support the hypothesis that mutations arising in the original poultry host would have been necessary for successful pathogen emergence in the novel finch host.

## RESULTS

Over the course of the experiment, HF1995 successfully established an infection in the tracheal mucosae of all 15 house finches inoculated, whereas Rlow established an infection in 12 of 15 (80%) inoculated birds (χ^2^ = 1.5, df = 1, *P* = 0.22). There was, however, a difference in the timing of the establishment of infection. At 2 days postinfection (dpi), HF1995-inoculated birds were significantly more likely to test positive for infection. M. gallisepticum could be detected in 12 out of the 15 (80%) birds inoculated with HF1995 but in only 6 of the 15 (40%) birds inoculated with Rlow (logistic regression: *z* = −2.2, df = 1, *P* = 0.03). By 7 dpi, however, all the birds that became infected (i.e., 15 HF1995 birds and 12 Rlow birds) tested positive for M. gallisepticum.

**FIG 1:**
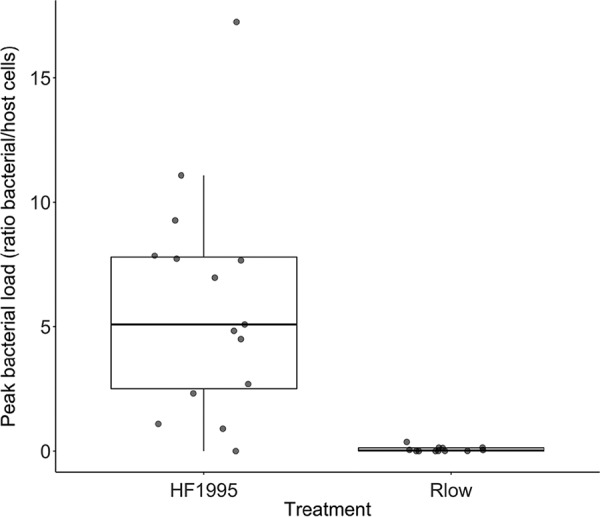
Boxplot diagram showing peak bacterial loads, estimated as the peak number of bacterial to host cells over the course of the experiment, in infected house finches following inoculation with either a poultry strain of M. gallisepticum (Rlow) or a house finch epizootic-outbreak isolate (HF1995). Boxplots show the median and range peak loads, with significantly lower peaks in birds inoculated with Rlow (*n* = 15; median = 0.025, range = 0.0006 to 0.37) than with HF1995 (*n* = 12; median = 5.09, range = 0.001 to 17.3). The dots show the raw values.

When considering only the birds that became infected, we found that HF1995-infected finches reached higher peak bacterial loads than Rlow-infected finches (Mann-Whitney U test: Wilcoxon test statistic (*W*) = 170, *P* < 0.0001) (Fig. 1). The number of days between inoculation and peak bacterial loads, however, did not significantly differ between treatments (Mann-Whitney U test: *W* = 104, *P* = 0.43; mean ± standard deviation: Rlow = 9.3 ± 4.6 days, HF1995 = 11.2 ± 6.4 days). By the end of the experiment (56 dpi), all 12 Rlow-infected finches had cleared the infection, whereas 3 of 15 (20%) HF1995-infected birds remained positive for M. gallisepticum (χ^2^ = 14.2, df = 1, *P* < 0.0002). Additionally, Rlow-infected birds cleared the infection significantly faster than HF1995-infected ones (linear model: *t* = −4.5, *P* < 0.001; mean ± standard deviation: Rlow = 22.8 ± 13.5 days, HF1995 = 41.5 ± 5.3 days).

**FIG 2:**
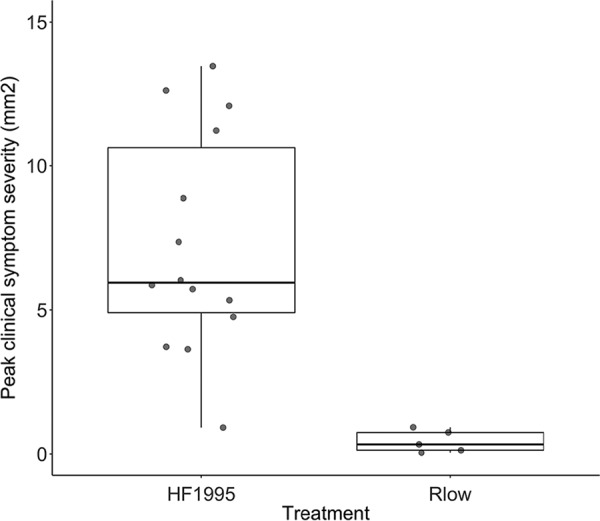
Boxplot diagram showing clinical symptom severity (in square millimeters) in infected house finches following inoculation with either a poultry strain of M. gallisepticum (Rlow) or a house finch epizootic-outbreak isolate (HF1995). Boxplots show the medians and ranges of conjunctival swelling, with significantly lower levels in birds inoculated with Rlow (*n* = 14; median = 0.33, range = 0.05 to 0.92) than with HF1995 (*n* = 5; median = 5.9, range = 0.9 to 13.5). The dots show the raw values.

While 14 out of 15 finches (93%) inoculated with HF1995 developed clinical symptoms (i.e., conjunctivitis), only 5 out of 12 (42%) Rlow-infected individuals exhibited conjunctivitis. This difference was significant. Rlow-infected individuals exhibited a significantly lower probability of developing clinical symptoms than those inoculated with HF1995 (logistic regression: *z* = −2.5, *P* = 0.01). Furthermore, when we considered symptomatic birds only, birds that were infected with HF1995 developed significantly more severe conjunctivitis than birds infected with Rlow (Mann-Whitney U test: *W* = 69, *P* < 0.001) (Fig. 2).

**FIG 3:**
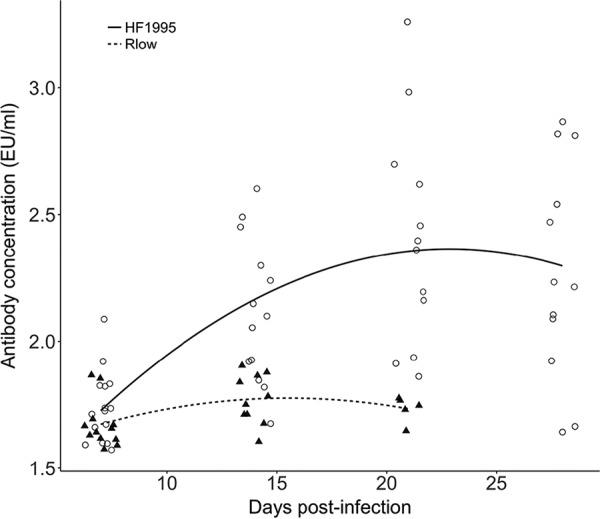
Circulating levels of specific anti-M. gallisepticum antibodies in infected house finches inoculated with either a poultry strain (Rlow) or a house finch epizootic-outbreak isolate (HF1995) over time (i.e., between 7 and 28 dpi); concentrations are reported as ELISA units (EU) per milliliter based on an arbitrarily assigned starting concentration of the undiluted pooled plasma sample used to create the standard curve. We show raw values of antibody concentrations in HF1995 (open circles)- and Rlow (filled triangles)-infected finches and best-fit regression lines.

Overall, there was a significant quadratic relationship between the production of M. gallisepticum-specific antibodies and time (linear mixed model; time: *F*_1,53.9_ = 35.8, *P* < 0.0001; time^2^: *F*_1,54.5_ = 8.2, *P* < 0.0001) (Fig. 3). However, the strength and pattern of this relationship differed significantly between the two treatment groups, leading to a significant treatment-by-time interaction (*F*_1,53.9_ = 8.2, *P* = 0.045). For example, while HF1995 triggered antibody responses to increase at a rate of 0.06 enzyme-linked immunosorbent assay (ELISA) units (EU)/ml between 7 and 14 dpi, Rlow did so at a rate of 0.01 EU/ml over that same period, generating a 130% increase in the amplitude of the response of HF1995-inoculated birds relative to Rlow-inoculated ones (treatment, *F*_1,58.8_ = 18.6, *P* = 0.022).

## DISCUSSION

If the host shift of M. gallisepticum from poultry to house finches had simply been the result of house finches coming into contact with infected chickens, then we predicted that a virulent strain of poultry M. gallisepticum, Rlow (31), should be able to successfully infect house finches upon inoculation. Contrary to this prediction, however, Rlow displayed a poor capacity to infect house finches relative to the 1995 house finch epizootic outbreak strain, HF1995. Indeed, compared to HF1995, Rlow achieved substantially lower bacterial loads following experimental inoculation, caused less severe clinical disease, and elicited weaker specific (i.e., antibody) immune responses in house finches. Additionally, house finches were able to clear Rlow much faster than HF1995. Taken together, our results indicate that contact with the novel host alone was insufficient to explain the host shift of M. gallisepticum from poultry and, instead, that a genetic change(s) was also necessary for emergence in house finches.

Poultry and house finch strains are known to exhibit a number of genomic differences (26, 33), but identifying the specific mutation(s) underlying the host shift is challenging. For instance, relative to Rlow, HF1995 (cross-listed as strain GA_1995 [26]) displays a reduced clustered regularly interspaced short palindromic repeat (CRISPR) diversity, numerous fixed, nonsynonymous single-nucleotide polymorphisms, and loss of 52 (or 8.6% of) known protein-coding genes through genomic deletions, disruption by novel insertion sequence elements, or mutations leading to pseudogenization (26). However, in light of our findings of a lower ability of Rlow to colonize the house finch respiratory mucosa and/or replicate within the novel finch host, a functional divergence in genes coding for proteins involved in cytoadhesion (33) is of particular interest. We know from numerous other bacterial host-pathogen systems that mutations in genes that influence adherence to the host mucosal epithelium and host cell uptake of intracellular bacterial pathogens impact disease progression (34–37). Accordingly, the attenuated poultry strain of M. gallisepticum, Rhigh, exhibits low levels of host cell adherence and minimal pathology in poultry relative to Rlow (38). This is thought to result, in part, from the loss of expression of *gapA* and *cmrA*, which encode proteins involved in cytadherence (32, 38). Consistent with dual roles of adhesin molecules, Rhigh is compromised in its ability to invade host cells, cross the poultry respiratory mucosa, and spread systemically (39–41). Other genes of potential interest include those that encode factors affecting metabolic capacity, which may play a role in the low pathogen load observed in this study. For example, transposon mutagenesis experiments revealed the metabolic enzyme dihydrolipoamide dehydrogenase, a subunit of a multienzyme involved in glycolysis, as a virulence factor in Rlow infection of poultry (42). Further experimental work examining bacterium-host cell interactions is required to fully understand the phenotypic changes associated with this host shift. Such work can help narrow the list of potential candidate genes responsible for the successful emergence of M. gallisepticum in the novel house finch host.

The finding that contact was not sufficient to allow M. gallisepticum to jump from poultry into house finches may explain why this pathogen, which is often found in other avian host species, seems unable to persist within these hosts (30, 43, 44). Infections of other passerine species are indeed thought to reflect spillover events from natural house finch host reservoirs (45). In support of this, a phylogenetic analysis of 107 M. gallisepticum strains from poultry, house finches, and other songbirds revealed that all isolates obtained from non-house finch songbirds clustered with house finch rather than poultry isolates (46). Furthermore, while house finch M. gallisepticum can infect a diverse array of passerines, it is pathogenic (i.e., causes conjunctivitis) only to closely related species within the family Fringillidae, such as purple finches (Haemorhous purpureus) and American goldfinches (Carduelis carduelis) (30, 43). Indeed, evidence for M. gallisepticum exposure, either via positive PCR-based detection of M. gallisepticum or positive antibody tests, was found in 27 species of wild birds representing 15 families, but clinical disease signs were rare or completely absent in species outside the family Fringillidae (29). Taken together, these studies suggest that transmission from house finches to other avian species occurs regularly as a result of contact but that contact alone is insufficient to enable M. gallisepticum to jump into any of these novel hosts. This is consistent with our findings, namely, that poultry M. gallisepticum is able to colonize mucosal surfaces of individual house finches but is somehow compromised in its ability to replicate, persist within, and/or cause pathology to the novel house finch host.

Our findings may also shed light on a phylogenetic study that found a singular M. gallisepticum strain collected from an asymptomatic house finch to be more closely related to poultry M. gallisepticum strains than house finch M. gallisepticum strains (46). Indeed, if contact alone was sufficient for M. gallisepticum emergence in house finches, then this occurrence should be more common, yet house finch M. gallisepticum strains examined to date have been shown to be derived from a single ancestor (26, 33). Given that the sampling of M. gallisepticum strains from house finches is both sporadic and conducted randomly, finding an M. gallisepticum strain closely related to poultry in house finches should be extremely unlikely unless spillovers of poultry M. gallisepticum strains into house finches through contact occur more frequently than expected. The unique outbreak of severe conjunctivitis in house finches attests to the fact that these spillovers are generally unsuccessful and that the genetic changes required for host shifts are themselves extremely rare.

## MATERIALS AND METHODS

### House finch capture, housing, and experimental infection.

We trapped male house finches at bird feeder sites in Alabama, USA, between August and September of 2014 (as described in reference 47). All birds used in the study were yearlings, having hatched in the spring of the calendar year in which they were collected. We collected birds from three sites in Auburn, AL, approximately 1.5 miles apart and from two sites in Birmingham, AL, separated by 8 miles. Upon capture, a blood sample (∼70 μl) and choanal swab were collected from each bird. Choanal swabbing consisted of inserting a swab into the bird's oral cavity and then swabbing the tracheal opening and choanal cleft for approximately 15 s. Blood plasma was used for a serum plate agglutination assay to test for anti-M. gallisepticum antibodies, indicating prior M. gallisepticum exposure (48). Swabs were used for PCR amplification of M. gallisepticum DNA to test for current infection (49). Birds positive by either test were immediately released and not retained for the experiment. The remaining birds then underwent a 30-day quarantine period, during which they were treated for infection by Trichomonas gallinae and Coccidia spp. Following quarantine, birds were randomly divided into treatment groups. Males in one treatment group (*n* = 15) were inoculated with an epizootic outbreak house finch M. gallisepticum strain cultured from a wild-caught house finch in Georgia, USA, in 1995 (HF1995 [passage 13]; cross-listed as GA_1995 in reference 26). Males in the second treatment group (*n* = 15) were inoculated with a poultry M. gallisepticum strain (Rlow [passage 17]), which was provided by Naola Ferguson-Noel (University of Georgia). We inoculated birds by dropping 10 μl of the respective M. gallisepticum culture into each eye, each containing approximately 1 × 10^4^ to 1 × 10^6^ color-changing units/ml of M. gallisepticum. To prevent M. gallisepticum transmission between treatments, we housed finches in separate rooms under identical conditions. Following inoculation, we monitored finches for the development of infection for 8 weeks (56 days), after which time all birds were humanely euthanized by CO_2_ narcosis in accordance with the rules established by the 2013 American Veterinary Medical Association Guidelines on Euthanasia. We also took a choanal (tracheal) swab sample on these days to test for the establishment of an M. gallisepticum infection and pathogen load using quantitative PCR (qPCR). All described work was approved by Auburn University's IACUC under PRN 2014-2517 and Biological Use Authorization 500.

### Quantification of clinical disease severity.

To document clinical disease signs (conjunctivitis), we photographed the right and left eyes of each bird, with the bird's eye parallel to the camera. We then quantified the area of the conjunctival swelling in the photographs using the programs TpsUtil version 1.46 and TpsDig version 2.16 (50, 51). Bill depth was measured with calipers to 0.1 mm. This measurement was then used as a scale in the images so that the area of conjunctival swelling in square millimeters could be assessed. The scaled picture files were then duplicated, with one file used for the placement of 10 landmarks around the inner ring of the conjunctiva. The duplicate file was used to place 12 landmarks around the outer area of the conjunctiva. Area measurements (in square millimeters) for the outer and inner rings of the conjunctiva were generated using TpsUtil. The area of the conjunctiva was then calculated as the area of the outer ring minus the area of the inner ring. To determine swelling severity, we subtracted the conjunctival area at day 0 (preinoculation) for a given individual from the area measured at a given sampling time point for that same individual. We estimated the background variation in our measurements by repeating this process with photographs of control birds. We used twice the average background variation as the threshold to determine whether birds displayed clinical conjunctiva swelling. The threshold value was subtracted from all measurements, with any values below the threshold being treated as having no, or zero, change in swelling.

### M. gallisepticum presence and load.

We tested the respiratory epithelia of all house finches for M. gallisepticum at 0, 2, 7, 14, 21, 28, 42, and 56 dpi. Choanal swabs were tested for the presence of M. gallisepticum via PCR followed by agarose gel electrophoresis (49). Briefly, swabs were placed in 100 μl of sterile nuclease-free water. Swabs were then placed at 100°C for 10 min, placed at −20°C for 10 min, and finally centrifuged at 13,000 rpm for 5 min. We tested the supernatant of each sample in duplicate for the presence of M. gallisepticum using the forward primer 5′ GCTTCCTTGCGGTTAGCAAC 3′ and reverse primer 5′ GAGCTAATCTGTAAAGTTGGTC 3′. PCR parameters were as follows: 94°C for 5 min, 35 cycles of 94°C for 30 s, 55°C for 30 s, and 72°C for 30 s, and a final 5-min extension at 72°C (49). In each assay, M. gallisepticum DNA extracted from pure culture served as a positive control.

For all M. gallisepticum-positive samples, we then quantified bacterial loads using the remaining choanal swab extract and a TaqMan qPCR amplification of the M. gallisepticum single-copy gene *mgc2*. To control for variation in the amount of starting material, we also amplified a house finch single-copy gene, *rag1*. The detection limit of this assay was previously reported to be less than 10 genomic copies (52). For HF1995- and Rlow-inoculated individuals, we confirmed the timing or lack of M. gallisepticum colonization by also performing qPCR on samples that were negative for M. gallisepticum at 2 and 7 dpi. To confirm M. gallisepticum clearance, we additionally ran qPCR on the sample collected after the last M. gallisepticum-positive sample for each bird. Before use, we cleaned up extracted swab samples using a Qiagen QIAquick PCR purification kit. All reaction mixtures were run on the ABI Prism 7500 system (Applied Biosystems). We made a standard curve of pooled genomic DNA to estimate the relative amounts of M. gallisepticum bacteria between individuals. We then divided the number of *mgc2* genes by one-half the number of *rag1* genes to approximate the ratio of M. gallisepticum cells (haploid) to host cells (diploid).

### M. gallisepticum-specific antibody (IgY) detection.

Comparisons of M. gallisepticum-specific antibody concentrations in plasma at 7, 14, 21, and 28 dpi were made using an enzyme-linked immunosorbent assay (ELISA) and a standard curve of pooled house finch plasma from experimental house finches. Briefly, the goat anti-passerine immunoglobulin Y (IgY) secondary antibody (53) was conjugated to horseradish peroxidase (HRP) using a Lightning Link HRP kit (Innova Biosciences) according to the manufacturer's instructions. Samples and standards were diluted in 1× sample-conjugate diluent (Affinitech, Ltd.), and then 100 μl of each was plated in duplicate onto M. gallisepticum-coated plates (Affinitech, Ltd.). After a 1-h incubation at room temperature (RT), plates were washed three times with 1× wash buffer (50 mM Tris-buffered saline, pH 8.0, with 0.05% Tween 20; Bethyl Laboratories). The HRP-conjugated antibody was diluted 1:10,000 in sample-conjugate diluent (50 mM Tris-buffered saline, pH 8.0, with 1% bovine serum albumin and 0.05% Tween 20; Bethyl Laboratories), and 100 μl of the diluted antibody was then added to each well. Plates were incubated for 1 h at RT and then washed three times in 1× wash buffer. One hundred microliters of the enzyme substrate 3,3′,5,5′-tetramethylbenzidine (TMB) one-component HRP microwell substrate (Bethyl Laboratories) was added to each well, and plates were incubated at RT for 15 min. The reaction was then stopped with 100 μl of ELISA stop buffer (0.18 M H_2_SO_4_; Bethyl Laboratories), and plates were read at 450 nm using a BioTek PowerWave XS plate reader. Samples were considered positive for M. gallisepticum-specific antibodies if the absorbance at 450 nm was at least three times the background. For the pooled standard curve, this cutoff was between dilutions of 1:6,400 and 1:12,800. All samples were run at the same dilution, allowing for comparison of concentrations between treatments based on the pooled standard curve but not determination of absolute concentrations. Because of this, antibody concentrations are reported as ELISA units (EU) per milliliter, with the starting concentration in the undiluted pooled sample being arbitrarily assigned.

### Statistical analyses.

All statistical analyses were conducted in R (http://www.R-project.org/). We tested for differences in the abilities of HF1995 and Rlow to establish an infection using a chi-square test. Differences in the probabilities that birds were infected at 2 dpi were determined using logistic regression, with infection status (infected/not infected) as the response variable and treatment (HF1995 or Rlow) as the explanatory term. Differences in the peak bacterial loads and in the times of clearance of the infection were modeled only in infected individuals by performing Mann-Whitney U tests, with either peak bacterial load or date of clearance as the response variable and with treatment as the explanatory term. We tested for differences in the abilities of HF1995- and Rlow-inoculated birds to clear infection using a chi-square test. Differences in the probabilities of developing clinical symptoms were modeled using logistic regression, with clinical symptoms (0/1) as the response variable and treatment as the explanatory term. We then investigated differences in the severity of conjunctivitis as a function of treatment in symptomatic individuals using only a Mann-Whitney U test, with peak conjunctivitis as the response variable and treatment as the explanatory term. To test for differences in circulating levels of anti-M. gallisepticum antibodies over time (i.e., between 7 and 28 dpi) in infected individuals, we used lme4 and performed a generalized linear mixed-model analysis, with antibody concentration as the response term and with time, treatment, and their interaction as explanatory terms; individual identity was specified as the random effect (54). All figures were made using ggplot2 (55).
